# The emergence of a suburban penalty during the 1918/19 influenza pandemic in Malta: The role of a marketplace, railway, and measles

**DOI:** 10.1371/journal.pgph.0002167

**Published:** 2023-09-01

**Authors:** Lianne Tripp, Lawrence A. Sawchuk

**Affiliations:** Department of Anthropology, University of Toronto, Scarborough, Ontario, Canada; University of Hong Kong, HONG KONG

## Abstract

The Malta 1918/19 influenza experience adds to our understanding of the pandemic by illustrating the importance of suburban populations, their vulnerabilities, and elevated mortality rates. Studies on the socio-geographical variation in the 1918/19 influenza mortality has largely overlooked the suburban experience, and thus the often-hidden heterogeneity of the disease experience is missing. A comparison of mortality rates across the three settlement types (urban, suburban, and rural) for the second wave of the pandemic revealed that there were significant differences across the settlement types (x^2^ = 22.67, 2df, p <0.0001). There was a statistically significant divide between suburban settlement type versus urban and rural communities. Further, the geographical division of the central suburban region had the highest mortality rate at 4.28 per 1000 living of all suburban regions. A closer examination of the central suburban communities revealed that the town of Birchicara was the driving force behind the elevated influenza mortality, with a rate of 5.28 per 1000 living. The exceedingly high rate of influenza mortality in Birchicara was significantly different from the other suburban communities (Z = 2.915, p = 0.004). Birchicara was notable as both a transmission and burden hotspot for influenza infection because of a unique conflation of factors not observed elsewhere on the island. Foremost, was the pitkali market, which was a produce wholesale distributing centre; second, was the fact that the train station was a central hub especially for Maltese labourers; third, was that the measles epidemic in 1916/17 contributed to elevated childhood influenza deaths because the presence of military personnel and their families. We argue that the interaction of the three factors, and in particular, the measles epidemic with childhood influenza, amounted to a syndemic. Factors associated with urbanization and high rates of infectious diseases, such as overcrowding and infant mortality, did not play a primary role in the syndemic.

## Introduction

The relationship of how cites can shape population health has long been an area of interest by scholars [1]. Throughout history, urban centres in Europe were observed to have features that were thought to contribute to poor health [2]. As such, during the 19^th^ and 20^th^ centuries, city dwellers were subjected to the conditions associated with the ‘urban health penalty’, because of the concentration of poor people, who were exposed to unhealthy environments leading to a disproportionate burden of poor health [1]. These ‘deathtraps of mankind’ subjected their inhabitants to crowded living conditions, poor sanitation, increased inter-personal contact, and at times, excessive mortality through acute infections. Yet, the mortality experience of urban dwellers was not homogenous and would vary because of a multitude of factors [3].

With respect to infectious diseases and urban centres, in the 19^th^ century, the high prevalence of such diseases was attributed to unsafe water, inadequate refuse management, poor food handling, crowded housing with poor ventilation, and commerce centres that were typically the entry point of diseases, especially in port cities [1,4,5]. Vlahov and colleagues [1] argue that the rate of urbanization may have been a more important predictor of population mortality, rather than the city size, because urbanization was an indictor of the extent of immigration into a population that was ‘outstripping resources’ [1,4].

The relationship of infectious disease outbreaks and urban living was commonplace regardless of the socio-economic status of the nation. In the 19^th^ century, and at the beginning of the 20^th^ century, urban centres in developed countries were decimated by infectious disease that were associated with crowded environments, and with limited sanitation. As such, epidemics of influenza, typhus, and tuberculosis killed millions of people [6]. Similarly, during the 20^th^ century, in ‘less wealthy’ countries, infectious diseases were the largest contributors to morbidity and mortality in city centres [6].

With the improvement of sanitation in many cities in both Europe and North America, in the mid 20^th^ century, there was an improvement in the quality of life and survivorship among those living in city centres. From an evolutionary perspective, changes in virulence of infections (e.g., scarlet fever) and progressively more immune (perhaps herd immunity) populations also contributed to improved health in cities [1,4]. By the later 20^th^ century, however, there again was a heightened poor health in urban centres relative to suburban and rural locations, which was attributed, in part, to the appearance of HIV in inner cities [6].

The relationship of urban city centres and infectious has not always been straightforward, however. In Medieval Denmark (1200–1400 AD), research on the prevalence of tuberculosis and leprosy found in skeletal remains indicated that there was not relationship to urbanity and other factors may be more important in explaining disease heterogeneity [7]. Further, exactly how the suburban environment factor into infectious disease prevalence is poorly understood. It is generally believed that suburban locations faired better than urban locations in terms of overall health, and that the health status of suburban populations fall somewhere between urban and the ameliorated health levels of rural environs [8]. Infant mortality rates indicate, however, that in terms of population health, suburban locations were diverse. In some suburbs, because of poor sanitation, many unincorporated outlying areas were as unhealthy as the worst city slums [8].

As the COVID-19 pandemic surpasses three years in most countries around the world, the geographical variation with respect to settlement types in COVID-19 rates have been observed. Higher COVID-19 morbidity and mortality rates have been observed in urban communities in China and the United States [9,10]. There have also been reports of elevated susceptibility in rural communities, where there are large elderly populations and because of limited access to health care [11]. The rates of COVID-19 in suburban communities have been largely overlooked in the academic literature, however. Elevated mortality COVID-19 rates in suburbia were apparent in Louisiana during the second peak of the COVID-19 when suburban rates were higher than the first peak as well as the rest of New Orleans, but comparable to the urban and rural rates [12]. In Canada’s most populous city, Toronto, the suburban communities were the hardest hit by the virus during the summer of 2020 [13]. Similarly, over hundred years ago, during the last ‘Great pandemic’ of 1918/19 influenza, the intensity of a pandemic varied greatly across settlement types, and the source of variation in regional patterns of influenza mortality remains a contentious point among scholars. The re-examination of the 1918/19 influenza pandemic mortality variation within the settlement type context has the potential to predict geographical variation of infectious disease during current and future pandemics, and to improve our understanding of why these diseases vary with respect settlement type.

Turning to island populations, in particular, small island populations, which provide unique epidemiological laboratories [14] of potential heightened vulnerability from both environmental and health factors [15]. With regards to the latter, the interconnectedness of insularity and health issues predominant in island studies on epidemics, famines, and quarantines [15]. The isolative nature of islands offer protection from infectious diseases, however, “it also makes the isolated population unusually vulnerable once the isolation is breached.” [16], (p. 23). Small populations are conducive to studying local distribution of disease without the interference of external factors that arise from interaction with peripheral communities [17,18], whereas geographical heterogeneity can be masked by aggregate nation-level data sets. The study of historic epidemics in small populations can enrich knowledge of the extent of the geographic heterogeneity and the variety of factors that influence the spread of infectious diseases across space and time [18].

During the 1918/19 influenza pandemic high mortality rates were observed in isolated island populations where approximately 8400 people or a fifth of the population of Samoa died, and approximately 22 percent of Western Samoans died from the disease [19,20]. Yet, other islands experienced low death rates, in the British Caribbean, among the islands (Bahamas, Barbados, Leeward islands, Jamaica and the Windward islands) had the highest influenza death rates at 1 percent [21]. Similarly, some Asian islands in the Pacific such as the Philippines experienced death rates between 0.68 to 0.92 percent, and in Indonesia the death rate was 3 percent during the fall wave of the pandemic in 1918 [22]. In Japan it was thought that 0.64–0.71 percent of the population died, but Chandra and Yu, 2015 [23] estimated that there was a pandemic associated population loss of 1.97 to 2.2 million people. Approximately 1.7 percent of the population died of pandemic influenza in Iceland [24], and similarly there was a 1.6 percent mortality among Indigenous population in Greenland [25], presumably low influenza death rates were experienced by the other islanders. Just under 6 percent died from 1918/19 influenza on the island of New Zealand from influenza, whereas approximately only 3 percent died in Australia; the average influenza mortality rate in in England and Wales was 0.27 percent, and the mortality rate in Newfoundland was 0.75 percent [18,26–28]. Others, such as America Samoa, New Hebrides, Solomon Islands, and Gilbert and Ellice Islands escaped the wrath of the disease altogether, because of effective quarantine of ships, or extreme maritime and socioeconomic isolation, in fact there was a 50-fold range in mortality rates across the Pacific islands [29,30].

Island populations, even small ones, experienced influenza death rates that were not homogenous across the island; on the Pacific islands, Indigenous populations experienced higher mortality rates relative to the smaller non-Indigenous residents [29,30]. There was also variation in spatial/geographical expression of mortality, and in particular with regards to rurality as a protective factor (for rurality having lower rates than urban areas, in other words, ‘the urban penalty,’ see: [26,31], and for rurality as not offering protection, ‘the rural penalty,’ see [32,33]), as well as heterogeneity in wave patterns, and in timing of the pandemic [29].

Explanations for regional heterogeneity of 1918/19 influenza patterns have focused on socio-geographic factors such as population size, population density, malnutrition, poverty, and overcrowded living environments, as well as sociopolitical factors [26,34–36]. A common observation among scholars is that there was a positive correlation between physical and social isolation (accessibility arising from geography, and/or lack of active transportation) and limited immunity to or lack of immunity to the virus [29,37]. The increased susceptibility to severe influenza and lethality owing to isolation is manifested in two different pathways: absence of the herald wave and/or limited exposure to previous influenza viruses or diverse respiratory pathogens (such as whooping cough or pneumonia), [18,29,38], or conversely that prior exposure to measles may have increased risk of lethal influenza because of the resultant diminished acquired immune memory loss, or ‘immune amnesia’ [39,40].

Regional suburban variation of 1918/19 influenza mortality has largely been neglected because this region was commonly lumped with the urban category or may have been non-existent in many locations [41]. The role of transportation, (ground, water, or air), in the transmission of the virus has been explored in the military [42] but has been examined to a lesser extent among civilian populations (for exceptions see: [43–46]). Contemporary studies on influenza and coronaviruses have concluded that air travel and airline transportation hubs are important for propagating infections, but the role of ground transportation is less clear [47].

This paper uses the case study approach to examine the 1918/19 influenza mortality patterns, in Malta, by administrative districts that were established by the colonial authorities, and by the various settlement types (rural, suburban, and urban), during the second wave of the pandemic, in the fall of 1918. To address the complex nature of the variation of 1918/19 influenza deaths on the island, we borrow the concept of the burden and transmission hotspots. In their study on hotspots, Lessler and coauthors [48], offer a clearer definition to geographic disease ‘hotspots,’ which has been has been mired in considerable ambiguity in meaning; further they propose that there are in fact three types of hotspots: burden hotspot, transmission hotspot, and emergence hotspot. In the case of a transmission hotspot, it is an area of elevated transmission efficiency, and burden hotspot is defined as “an area of elevated disease prevalence or incidence or a geographical cluster of cases” [48] (p. 1271).

First, we explore socio-geographic factors for regional heterogeneity in 1918/19 influenza deaths, with regards to settlement type (e.g. urban, suburban, and rural) such as population density, and infant mortality. The impact of the railway in increasing risk of influenza deaths is also explored.

Second, we assess the impact of deaths from epidemics of childhood infections (measles and whooping cough) on influenza deaths in children under 15 years of age by settlement type. While previous studies have alluded to the to the potential for measles to induce immune amnesia in subsequent infectious disease epidemics. [49], this study is the first to empirically test this association. We seek to determine whether there was a syndemic of measles or whooping cough with childhood infections with influenza under 15 years of age. A syndemic is “The comorbidity of two of more diseases or health conditions in which there is interaction of the diseases that “exacerbates the negative health effects of any of all of the diseases involved.” [50] (p. 941). Although the concept has arguably evolved over the last twenty years, two constants remain. First, “noxious social conditions” [51], (such as social inequality, whether poverty, overcrowding, stress or stigma) precipitate increased disease clustering or physical or behavioral vulnerability [50]. Second, as a result of the synergistical interaction of two or more epidemics [or health problems], there is an “excess burden of disease in a population” [52] (p. 425).

### The study population: Background

#### Overall health and influenza epidemic in Malta

During the early 20^th^ century, the standard of living in Malta was considered to be substandard relative to much of Europe. The health and sanitary infrastructure during the study period was uniformly abysmal and background living conditions were largely homogenous. The majority of the Maltese consisted of the working poor. During the study period, Malta was characterized by overcrowding, high unemployment rates, large family size, nutritional stress, inadequate public and private sanitation. The overall life expectancy at birth for the Maltese from 1911 to 1924 was just 44 years [53], which was considerably lower than other European locations. For example, in 1900 the overall life expectancy at birth for England and Wales was approximately 48.25 years, whereas Malta was 40.65 years [54]. Further, another indicator of overall health, the infant mortality rate (IMR) was extremely high throughout the early part of the 20^th^ century in Malta. From 1900 until 1940, Gibraltar (another British colony also considered to be a place with a poor standard of living and health), had consistently lower infant mortality rates that were approximately 100 infant deaths per 1000 live births lower than the rates in Malta. For example, in 1918, Malta’s infant mortality rate was just shy of 250 infant deaths per 1000 live births, whereas Gibraltar’s infant mortality rate was 120 infant deaths per 1000 live births [55]. From 1929 until 1933, Gibraltarian males enjoyed a 15-year life expectancy advantage over Maltese males, whereas Gibraltarian females enjoyed a 23-year advantage over the Maltese females [55]. Most of the differences in survivorship between the two colonies in life expectancies arose in the 1 to 4 years of age categories. These differences in survivorship could be accounted for, in part, because of scale differences in territory and population size, with Malta’s population of over 100,000 being dispersed across the island it would have been more challenging to meet the health needs compared to the more concentrated population of under 20,000 within the town portion of Gibraltar [56]. The differences in fertility (birth rates were 23.8 births per 1000 living in Gibraltar compared to 33.7 per 1000 living in Malta) can be explained by the differences in survivorship and in particular the infant mortality rates. Finally, the contribution of the disproportionately high rates of childhood mortality in the rural communities of Malta [55] greatly contributed to the overall lowered survivorship in Malta during the early part of the 20^th^ century.

The influenza epidemic first appeared in Malta at the Cala Frana Seaplane Depot in June 1918, which resulted in 31 cases [57]. Among the civilian population, cases emerged in July 1918 until August 1918, as a mild uncomplicated infection scattered across six communities (Senglea, Silema, Luca, Tarxien & Paola, Valletta, and Zeitun) on the island. As reported by the Principal Medical Officer of Health, Dr. Critien, the herald wave claimed 34 civilian lives [34]. The second wave began in mid-August mainly among the patients and staff of St. Elmo hospital and among the 1^st^ G.B. Northumberland Fusiliers at Polverista Barracks (Cospicua) [57]. Among the civilians, the first recorded cases of the second wave were among the working classes in Zeitun in September 1918 [57]. Overall, the 1918/19 influenza mortality rate (for both waves) for Malta was 0.39 percent, which was similar to many other low rates of 1918 influenza deaths observed on island populations around the world. Previously, it was reported that on the smaller island of Gozo, children under the age of 5 years, introduced the virus to households and infected female adults at higher rates than their male counterparts [58]. On the main island of Malta, there was evidence of a syndemic effect of respiratory tuberculosis with pandemic influenza during the height of the pandemic, in the month of October. This syndemic relationship was not observed in Gozo [58].

**Fig 1:**
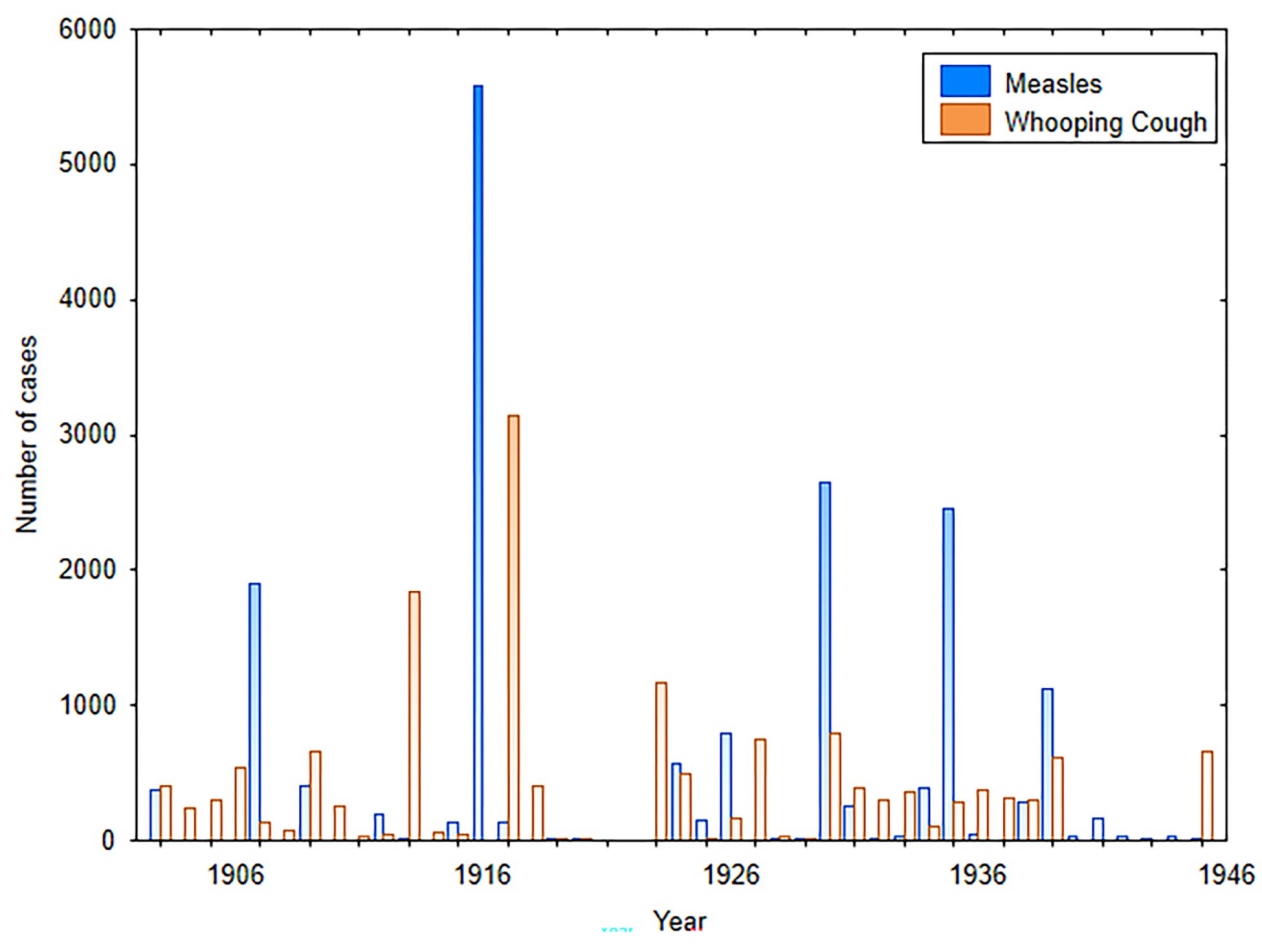
Measles and whooping cough epidemics from 1903 until 1946.

**Fig 2:**
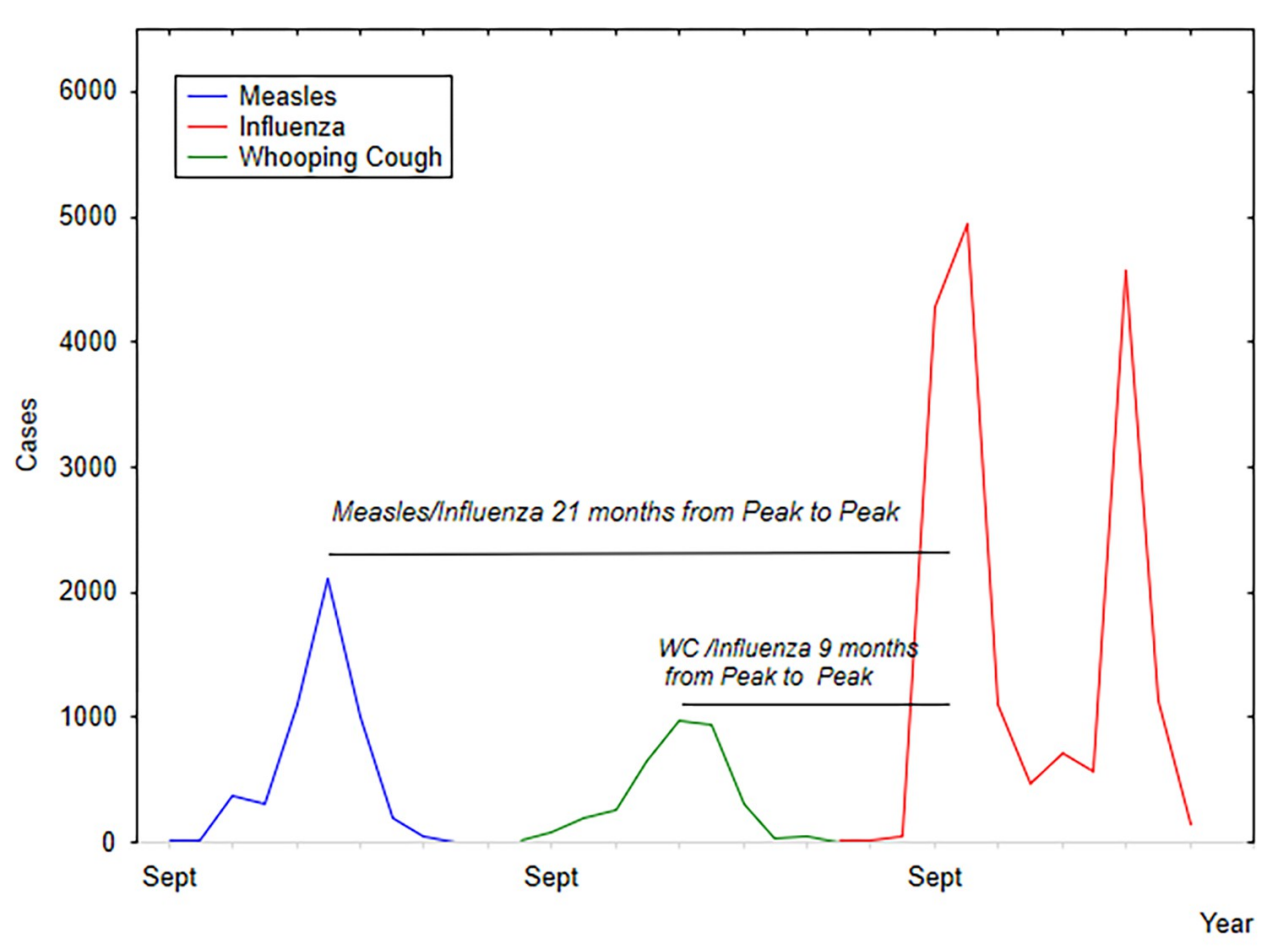
Timing of childhood epidemics of measles, in 1916/17, whooping cough 1917 and the 1918/19 influenza pandemic.

Prior to the 1918 pandemic, Malta experienced two childhood epidemics of note. First, in July 1916, there was an outbreak of measles which was not endemic to the island, but would appear in epidemic form at intervals of three to five years (e.g., 1903; 1909/10; 1911/12), this sporadic occurrence was similar to other island populations (see: [59–64]). The epidemic peaked in January 1917, and extended over a 14-month period (see Figs 1 and 2).

The 1916–17 measles event was unusual in that a large proportion of the cases were older children, relative to previous epidemics, with 45 percent of the cases occurring in children 5 years and older on the island of Malta [65,66] (see Table 1).

**Table 1:** Number of cases and deaths during the 1916–17 measles epidemic* [65,66].

Age period	Number of cases	Number of Deaths	Case fatality rate per 100
All ages	5572	235	4.22
0	374	48	12.83
1	735	95	12.93
2	668	31	4.64
3	564	20	3.55
4	620	4	0.64
5	2074	31	1.49
10	316	3	0.95
15	105	2	1.905
20	66	0	0
25	38	1	2.63
35 & upwards	12	0	0

*The Health Report covers an administrative period from April, 1916 to end of March, 1917. The Data presented in this Table covers both Maltese Islands, Malta and Gozo.

A total of 5572 measles cases were recorded with 235 deaths. The Medical Officer of Health stated that that the actual number of cases was not reflected in the recorded number. Second, in 1917, there was an epidemic of whooping cough, in Malta. Whooping was endemic to Malta, and at times was exacerbated at irregular intervals by epidemics. In September, the epidemic of whooping cough spread to all the settlements on the island. A total of 2854 cases were notified between October 1917 to May, 1918, along with 215 deaths.

#### Economy and the First World War

During the first three decades of the 20^th^ century, Malta’s economy was underdeveloped. The growth of the Maltese economy depended principally on providing services for the Royal Navy and British [67]. Specifically, Malta’s topography with its marginal *Xaghri* (*karstic* land) and the overall scarcity of productive land meant that the population of Malta was largely dependent on food import and attracting military expenditure from the colonial ruling power for their well-being. It has been argued that because the needs of the British military were top priority, the citizens of Malta were neglected and resulted in misgovernment, thus fueled not only mistrust of the governance, but poverty of the Maltese [68,69]. In fact, the island was viewed by the Governor of Malta, Admiral Sir John Fisher as “ha[ving] no value whatsoever. It produces nothing. It has no manufacturers… Malta has no military value whatever, it exists for the Navy, and exists by the Navy.” [70]. Politically, the Maltese did not have any autonomy over governance, and local affairs including the health care system.

As a strategic stronghold for the British Armed Forces, Malta during the First World War, served as supply and refitting station for the British military, as well as having dockyards that were important locations for the British Navy because of the proximity to Italy. Military barracks and hospitals were scattered throughout the Maltese islands putting civilian and the military in close and constant contact with each other.

The presence of military became even more pronounced during the First World War when Malta assumed the status of the’ Nurse of the Mediterranean.’ Not surprisingly then, the economy of island of Malta during the course of the war was dependent on providing services for the Royal Navy [69]. As the war progressed, there was a downturn in war related activities, which culminated in a large number of unemployed men and women. While the war effort initially brought economic prosperity to the Maltese, the prosperity was short lived. By 1916, the cost of the living, doubled; unemployment rates, employment instability, and food shortages rose [71,72]. In addition, to the rise in the cost of food, the food that was available, was inferior in quality and difficult to purchase [73]. During the war, the price of wheat increased and the availability of flour decreased significantly, to the point where the price of bread trebled by the end of the war. The significance should not be lost because bread was the staple food and the major source of energy for the poor, which made up a majority of the Maltese people.

The Maltese were not directly involved in the war as combatants but rather served in the Labour Battalions which played an indispensable role in sustaining logistical and line of communication support for frontline troops [74]. The majority of men were deployed abroad, for short periods, from a few months to a year, to locations such as Gallipoli, Egypt, Cyprus, Salonica, Taranto, or Turkey. They were recruited by the Maltese Labour Corps to work as dockhands and construction workers, or enlisted in the Royal Malta Artillery and Kings Own Malta Regiment, or the Royal Navy and Royal Navy Reserve. Up to 20 percent of the male Maltese population ([75]; approximately 15, 000 men) served in the war; we estimate that only half of these men (approximately 7500 men) served overseas [74,76–78]. The total population under British military command at Malta was estimated at 14,500. This consisted of a garrison averaging 7,400 troops, about 4,000 patients of the Expeditionary force in the 11 hospitals scattered through Malta. The remaining numbers included the Royal Air Force, Prisoners of War (2,100 in number), women, children, and Red Cross personnel employed by the Army and Navy.

#### Desirable research properties

British colonial Malta during the 20^th^ century had a number of desirable research qualities that are not found in larger nation state societies where inherent heterogeneity of population features are the norm, and potential confounders can not be controlled.

First, the possibility of variation in influenza rates because of difference in religious practices can be eliminated as a source of variation because the population as a whole, followed strict Roman Catholic teachings. This meant that across the island (as well on the smaller sister island of Gozo) mass gatherings associated with religious observations were similar, with the exception that each town had their own designated feast.

Second, the population was geographically and culturally isolated, and lacked any economic opportunities, which provided little incentive for any significant immigration to the Maltese islands. In large nation states such as the United States, immigration at the turn of the 20^th^ century was a dominant disruptive demographic feature. Any disturbance arising from immigration can be ruled out as a potential source of disease introduction.

Third, under British colonialism, and as an island nation, the Maltese never developed sufficient food supply, and were highly dependent on food imports to maintain adequate nutritional needs. Much of the economy was driven by providing for the British Imperial needs as a strategic colony. Consequently, the war period was a major stressor as food prices rose; by 1916, the cost of food was unaffordable even for the higher classes, and monopolization of staple foodstuffs, such as bread and meat exacerbated the situation [71,73].

## Materials and methods

Malta is rich in demographic information owing to its former British colonial status (from 1814 until Sept 20^th^, 1964) that follows in the tradition of excellent record keeping.

**Fig 3:**
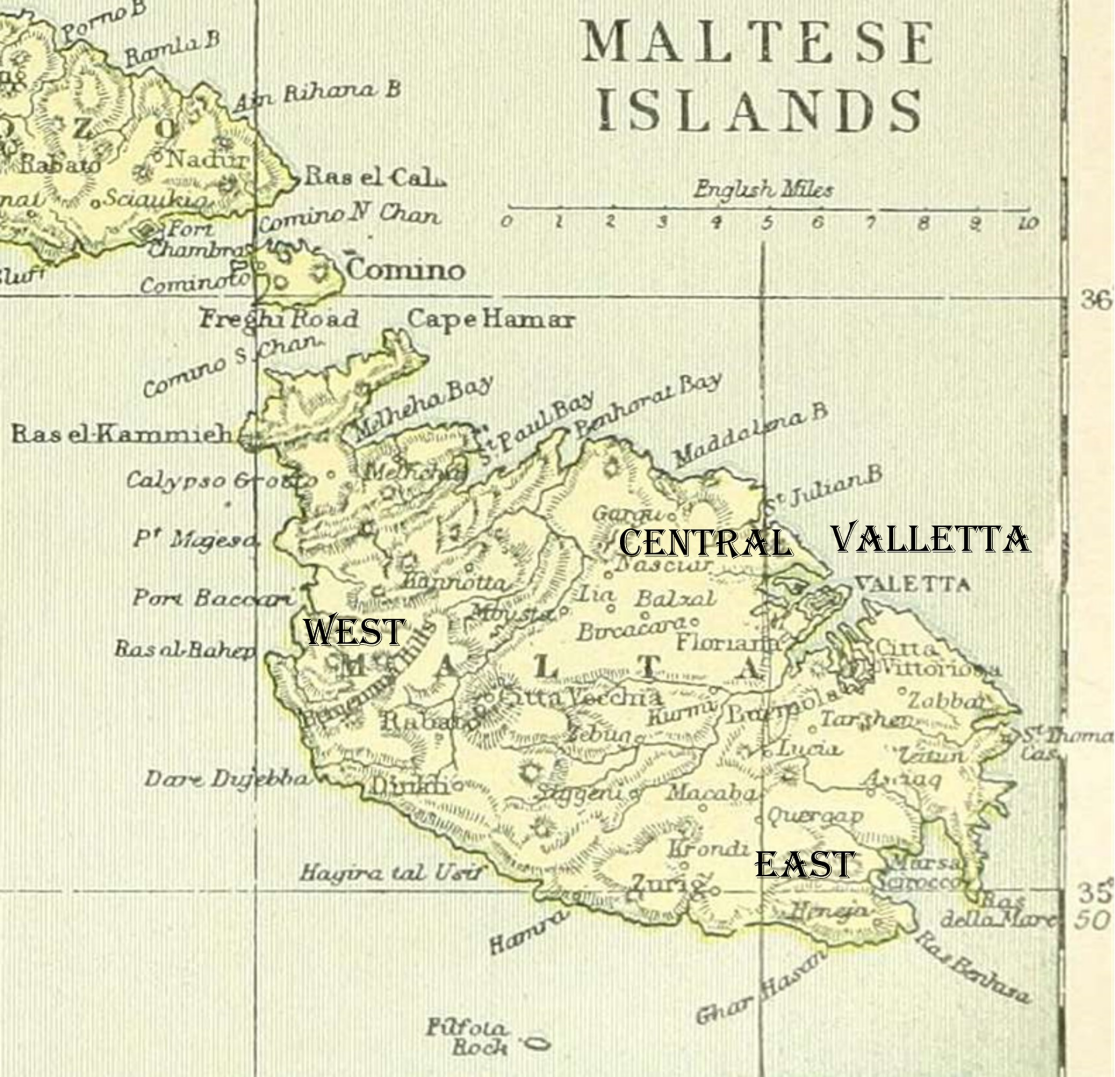
Map of Malta from 1892 showing the four districts of Malta and the location of each town and city (This map is copyright free map and can be located at: https://commons.wikimedia.org/wiki/File:Map_of_Malta,_The_Encyclopedia_britannica,_1892.jpg).

Identification of settlement type (either, urban, suburban, or rural) for each of the thirty-one towns and cities (there were 39 towns, but some locations were grouped together because of their proximity to each other, and small population size as set out in the censuses) was determined by the categorization described in the 1911 and 1921 censuses [79,80]. There was not any change in settlement type classification between the two census periods. Information on geographical location of the towns and cities in the 1911 census [79] was delineated by district: Valletta, East, West, and Central (see Table 2, and Fig 3). By combining the information on settlement types with district types, we created eight geographical divisions: Valletta urban, Valletta suburban; Central suburban, Central rural; Eastern urban, Eastern suburban, Eastern rural; and Western rural (see Table 2).

**Table 2:** Eight geographical divisions showing population and influenza mortality information for their respective towns and cities.

District/region	Town/City name	Number of Influenza deaths	Population Size	Influenza Mortality rate
*Valetta Urban*				
	Valletta	51	24128	2.11
	Floriana	10	6478	1.54
Total		61	30606	1.99
*East Urban*				
	Cospicua	37	12681	2.92
	Vittoriosa & Calcarra	16	8063	1.98
	Senglea	20	8795	2.27
Total		73	29539	2.47
*Valletta Suburban*				
	Sliema & St julians	50	17004	2.94
	Misida & Pieta	10	5307	1.88
	Hamrun	60	15584	3.85
Total		120	37895	3.17
*Central Suburban*				
	Birchicara	52	9855	5.28
	Curmi	34	10218	3.33
Total		86	20073	4.28
*East Suburban*				
	Zabbar	16	7341	2.18
	Tarxien & Paula	20	7591	2.63
Total		36	14932	2.41
*West Rural*				
	Siggieui	1	3580	0.28
	Rabato & Notabile	36	9663	3.73
	Dingli	1	1054	0.95
	Musta, St. Paul & Miaggaro	3	8380	0.36
	Melleha	9	3021	2.98
Total		50	25698	1.95
Central Rural				
	Lia, Attard, & Balzan	6	5069	1.18
	Naxaro	15	3261	4.60
	Gargur	3	1563	1.92
	Zebbug	12	6238	1.92
	Luca	7	4050	1.73
Total		43	20181	2.13
East Rural				
	Marsascirocco	2	715	2.80
	Micabiba	4	1410	2.84
	Zeitun	35	9060	3.86
	Asiak	2	1908	1.05
	Gudia	2	1257	1.59
	Zurrico	9	4772	1.89
	Chircop	0	814	0.00
	Safi	0	434	0.00
	Crendi	4	1620	2.47
Total		58	21990	2.64

Because the majority of influenza deaths (66.2%) occurred during the second wave (during the months of September to November 30^th^, 1918), the study was restricted to the second wave. To examine the overall pattern of influenza mortality on the island of Malta, the weekly death rates were estimated by using death information provided in the Government nominal Death Registers for the year 1918 [66]. In addition to providing details as to name, age at death, place of birth, place of residence, name and surname of parents whether living or dead, the cause of death was used to generate the influenza mortality rate. The death data was extracted and transferred to an Excel file. Causes of death identified as influenza or influenza complication (pneumonia or broncho-pneumonia), were included in the weekly death rates per 1000. The 1911 census for the Maltese Islands [79] provided information on the baseline for the reconstruction of the population at risk for the pandemic study period.

Weekly counts of influenza, pneumonia, or broncho-pneumonia deaths for each of the settlement types (urban, suburban, rural) were tallied and divided by the total population size for each settlement type. The Annual Heath Report for 1918/19 yielded estimated population size for each community for the year 1918 [81], the weekly rates were multiplied by a radix of 1,000.

The k-proportion test and two proportion test in XLSTAT [82] was used to compare proportions of influenza deaths across the three settlement types, and by the eight geographical divisions. In particular, the Monte Carlo method was used to compute a distribution of the x^2^ distance through a large number of simulations (N>15,000). A comparison of all pairs (settlement type and division) of proportions (total number of influenza deaths during the second wave/population at risk) was assessed via the Marascuilo procedure to identify settlement types and divisions that had significantly higher proportion of influenza deaths. The Marascuilo procedure compares all pairs of proportions, which enables the proportions possibly responsible for rejecting H_0_ to be identified. The Marascuilo procedure is recommended only if the chi-square test or the equivalent test based on Monte Carlo simulations reject H_0_ [83].

Kruskal-Wallis H test, also known as one-way ANOVA on ranks in SPSS [84], was used to assess differences in tempo in the weekly influenza rates across the three settlement types (urban, suburban, and rural, as well identify any towns or cities that had elevated rates of influenza rates) over the duration of the second wave.

To examine whether prior infections of childhood diseases such as measles and whooping cough deaths may have been a factor in elevated influenza rates, information on the two diseases by settlement were extracted from the Annual Health Reports of 1916 through 1918 [81,85,86]. The underlying assumption is that death certification from either measles or whooping cough was a better measure of its occurrence in the population than notification of morbidity data. The Medical Officers of Health in Malta frequently stated that the number of cases of infectious diseases such as measles and whooping cough were an underestimate (see [85]). Underreporting was a byproduct of: (1) the public indifference to mild cases of infectious diseases that were commonplace because the infection usually would pass without serious consequence; (2) the inconvenience and cost of reporting infectious diseases to a physician; and (3) the inability to record all cases during the height of the epidemic because the number of cases would swamp the capabilities of the limited number of physicians on the island. The world view in Malta regarding childhood survivorship was greatly influenced by frequent visitations of disease, lack of clean water, undernutrition, and the fact that infant and childhood mortality was typically 25 percent or higher [55].

Z-score tests in SPSS [87] were used to assess differences in the mortality rates by location during the epidemics of measles (1916–1917) and whooping cough (1917). Graphs were created using the program Statistica Version 10 [88].

Multiple regression was used to assess the influence of measles deaths, whooping deaths, and suburban settlement type in predicting the variation in childhood (under 15 years of age) influenza deaths (outcome variable) distribution. To normalize the distribution of the total number of influenza deaths under 15 years, a square root transformation was applied. The Kolmogorov- Smirnov test confirmed the transformed the data was normally distributed, because the p-value was non-significant (p = 0.089) [87]. A two-proportionate analysis for measles and whooping cough for suburban versus the two other settlement types was performed in XLSTAT [82].

## Results

**Fig 4:**
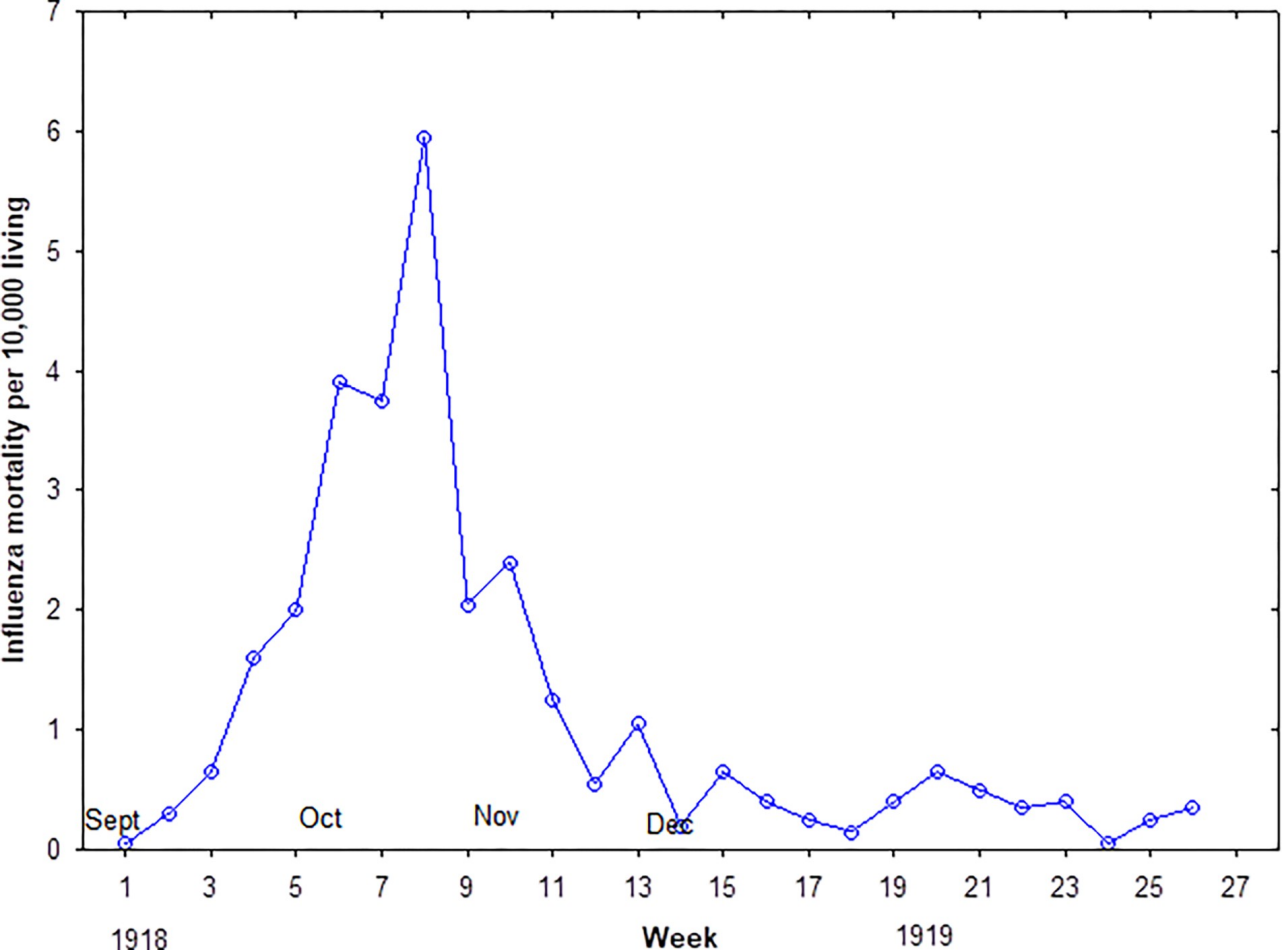
Influenza mortality rates among the Maltese during the second and third waves of the pandemic.

The second wave of 1918/19 influenza began in September, peaked in mid-October and subsided at the end of November. The third wave was modest in mortality and peaked in March (See Fig 4).

Table 2 shows influenza mortality rates during the second wave for each town/city according to district and settlement type (8 geographical divisions). The location of each town and city is indicated on the 1907 map (Fig 4). Unexpectedly, the highest rate of 1918 influenza mortality during the second wave was observed in the central suburban area at 4.28 deaths per 1000 living, and the suburban city of Birchicara had the highest rate at 5.28 deaths per 1000 living. The only other community to have experienced an influenza mortality rate close to that of Birchicara was that of Naxaro at 4.60 per 1000, however the small population size of about 3000 citizens means the rate may be susceptible to stochastic error. A K-proportionate analysis, using the Monte Carlo method, for the proportion of influenza deaths showed that there were significant differences across the geographical divisions (x^2^ = 45.756, 7df, p < 0.0001). The only significant differences across the eight divisions that met the critical value of 0.001 for the Marascuilo procedure for pairwise comparisons were: Valletta urban versus Central Suburban, Central Suburban versus East Suburban, and Central Suburban versus West Rural.

The initial analysis of the aggregated settlement types of urban, suburb and rural resulted in a clear significant disparity with the highest rate observed in the suburban communities at 3.32 per 1000 versus the much lower rates in the urban (2.23 per 1000) and rural communities (2.17 per 1000) (see Table 3).

**Table 3:** Wave 2 Influenza mortality rates for the three settlement types in Malta.

Settlement type	Number of settlements	Number of Influenza deaths	Population size	Average Influenza mortality rate (per 1000)	Range of Influenza mortality rates for each settlement type (per 1000)	
					Highest rate	Lowest rate
Urban	5	134	60145	2.23	1.96	2.29
Suburban	7	242	72900	3.32	2.75	4.33
Rural	19	147	67869	2.17	1.53	2.72

K-proportionate analysis of the proportion of influenza deaths showed that there were significant differences when analyzing by settlement type with the Monte Carlo method, yielding an overall chi square of 22.67, 2df, p <0.0001. Further, the Marascuilo procedure for pairwise comparisons showed that there was not a significant difference between urban and rural settlements (critical value less than 0.001), which was contrasted by a sharp and statistically significant divide between the suburban settlements versus the other areas (see Table 4).

**Table 4:** Marascuilo procedure for pairwise comparison of influenza mortality across the three settlement types.

Pairwise comparison	Critical Value*	Significant
Urban vs. Suburban	0.001	Yes
Urban vs. Rural	<0.0001	No
Suburban vs. Rural	0.001	Yes

x^2^ = 22.67, p>0.0001

*critical value must be 0.001 or greater.

A z-sore test for two proportions (between Birchicara and the rest of the suburban communities) confirmed that Birchicara was the driving force behind the observed disparity between suburbs with the other two settlement types (Z = 2.915, p = 0.004).

**Fig 5:**
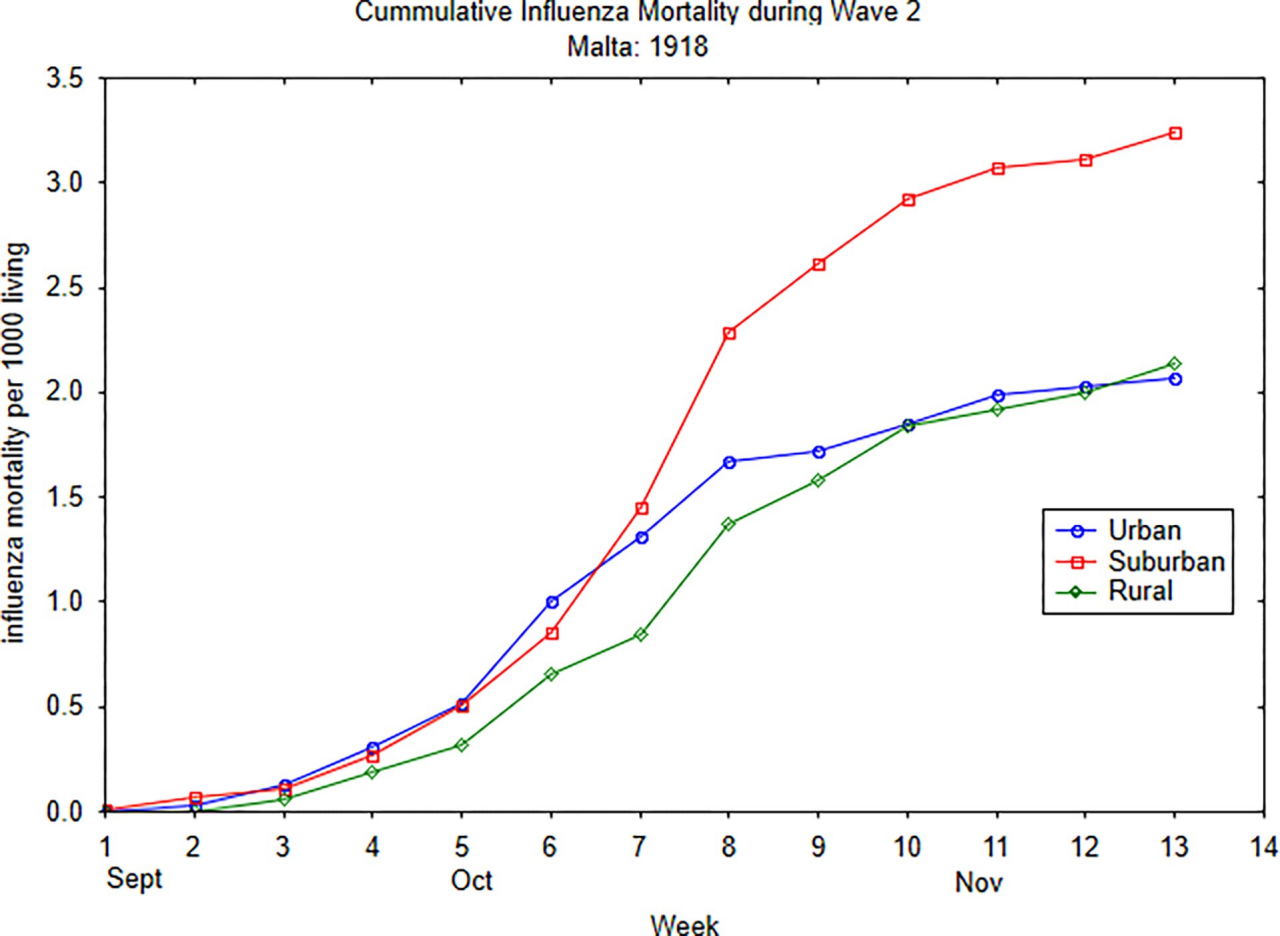
Weekly cumulative influenza mortality rates by settlement type (urban, suburban, and rural) during wave 2.

**Fig 6:**
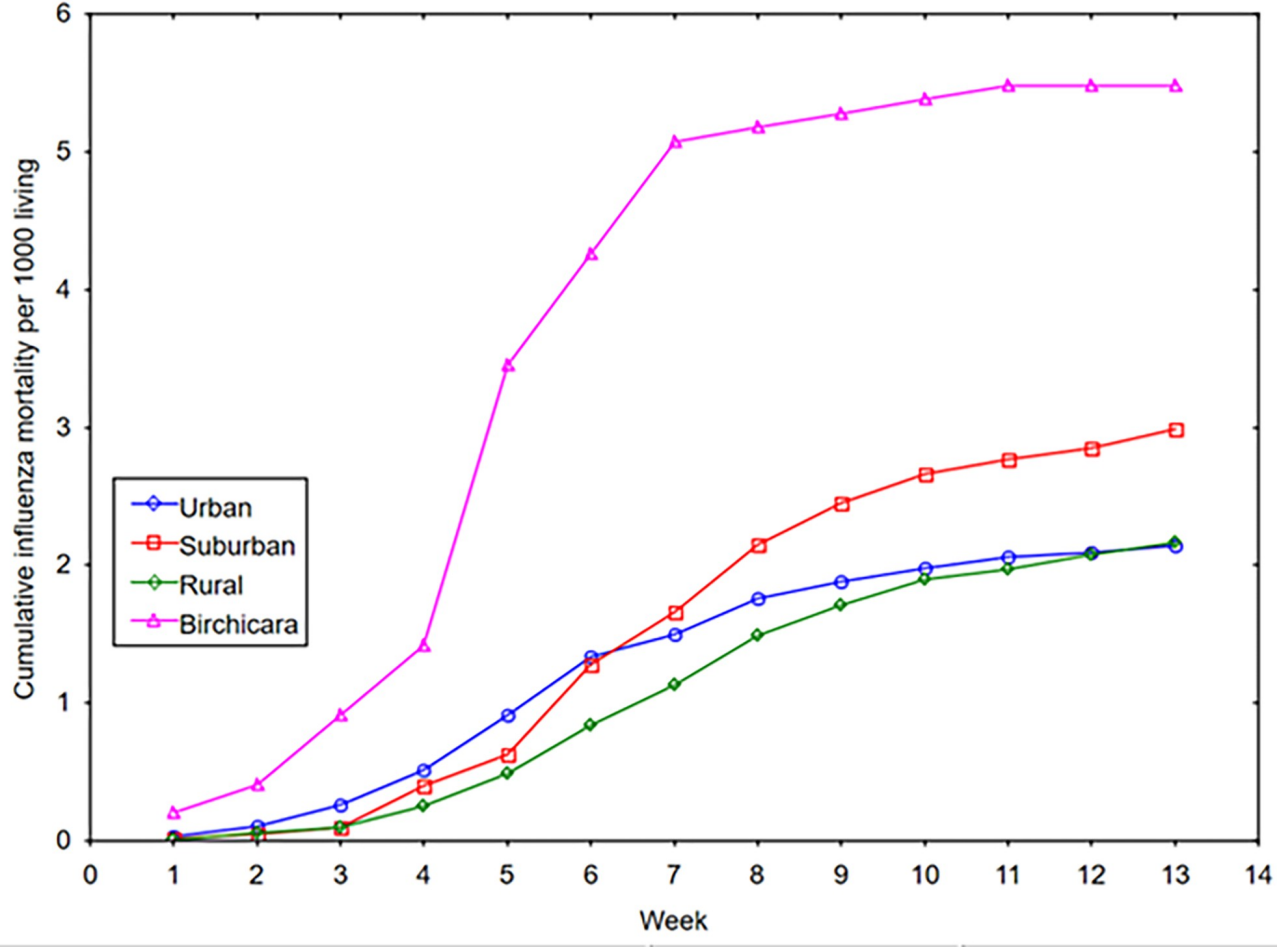
Weekly cumulative influenza mortality rates by settlement type and for Birchicara during wave 2.

The weekly cumulative influenza mortality for the rural and urban settlement types appeared to have a similar progression, whereas the tempo of influenza deaths in the suburban settlement type diverges from the other two settlement types and steadily increased by week 7 (see Fig 5), As shown in Fig 6, however, the cumulative influenza mortality of Birchicara was the driving force in the disparity of rates between the suburban with rural and urban settlement types. With cumulative rate for Birchicara removed from the rest of the suburban settlements, it is apparent that Birchicara showed a significant departure from the relative homogeneous tempo of the three settlement types for the weekly deaths during the 13 weeks of wave 2 of the pandemic (Kruskal Wallis-Wallis H = 10.93, df = 3, p = 0.012). The three settlement types: urban, suburban, and rural showed a common progression of deaths (Fig 6). As early as week 2, (Sept 8^th^ to 14^th^), the tempo of influenza mortality increased in Birchicara, and thereafter there was a rapidly increase in mortality rates until week 7 (Oct 13^th^ to 19^th^), followed by a slight increase in mortality during the remaining weeks (Fig 6).

### Measles & whooping cough

Birchicara experienced a significantly higher measles death rate than the other settlements with a rate of 2.73 per 1000 living as opposed to a measles death rate 0.96 per 1000 living for the rest of the island, (Z = 3.20, p = 0.0001). With respect to whooping cough, Birchicara experienced a significantly higher death rate than the other settlements at 1.62 per 1000 living as opposed to 0.700 per 1000 living, (Z = 2.11, p = 0.035).

The overall regression model of whooping cough deaths, measles deaths, and suburban settlement type for explaining influenza deaths under fifteen years of age, was significant (F = 12.242, df = 3, p = < 0.001). The categorical variable, suburban, was contrasted with urban and. The adjusted R-square shows that 53.8 percent of the variation in the influenza deaths <15 (transformed) were explained by two predictors: measles (t = 2.85, p = 0.008), and suburban settlements (t = 2.12, p = 0.043). Whooping cough was not a significant predictor (p = 0.610; see Table 5). The standardized beta coefficients show that in relative terms that measles (B = 0.645) had a larger effect on childhood influenza deaths than settlement type (B = 0.345; see Table 5).

**Table 5:** Regression output for influenza under 15 years of age*.

	Standardized coefficients Beta	t	p-value
(Constant)		-0.276	0.785
Suburban	0.345	2.124	0.043
Measles deaths	0.645	2.848	0.008
Whooping cough deaths	-0.129	-.517	0.610

* The dependent variable is the square root of influenza deaths under 15 years of age.

A z-score test assessing the two proportions of measles deaths between the suburban settlement type and the two other settlements combined (rural and urban) confirmed that suburban settlement had a higher proportion of measles deaths relative to rural and urban settlement types (Z = 2.120, p = 0.034).

## Discussion

The discovery that the central suburban area, let alone the city of Birchicara, had the highest influenza mortality rate during the height of the pandemic in Malta was unexpected. This finding challenged conventional reasoning that large urban centres were where the influenza pandemic took hold and would have the highest rates. For example, the capital of Valletta, was expected to have experienced the greatest toll from the pandemic, especially since the dockyard was in full operation providing aid to the British Navy during the First World War. Many Maltese men from across the island were employed as dockhands and intermingled with the British service men, and yet the influenza rates (see [58] for morbidity rates) were not excessive in Valletta during the second wave of the pandemic.

An investigation into the factors that contributed to the elevated mortality rate in Birchicara, revealed that a unique set of circumstances created an environment that was conducive to the transmission of the virus which resulted in the highest mortality rate on the island, one that can be argued to have represented a ‘suburban penalty.’ We propose that Birchicara was both a ‘burden’ and ‘transmission’ hotspot [48] for influenza infection because of a unique conflation of factors not observed elsewhere on the island. Foremost, was the pitkali market, which was a produce wholesale distribution centre, and the largest on the island; second was the fact that a train station was located in the city of Birchicara and was a central hub and destination especially for Maltese labourers; and third was that a recent epidemic of measles which was introduced or facilitated by the military presence and their families in Birchicara, increased the susceptibility to the influenza virus. The interaction of these three factors resulted in a syndemic of measles with pandemic influenza in Birchicara that was not observed elsewhere on the island.

### The pitkali market: Produce market wholesaler

The primary factor for influenza transmission and the resultant excessive mortality, and what set Birchicara apart from other settlements was that it was the location for the largest wholesaler market of fruits and vegetables on the island. A review of the literature on marketplaces that have been implicated as the source of epidemics has revealed that the focus has been on food-borne infectious diseases originating in wet or meat markets (see for example wet market and COVID-19 [89,90]).

Otherwise known as *pitkali*, the wholesale market was located near the railway station gardens, on the main road and it was also the Tram terminus. According to Paul Galea, a local expert on the Maltese railway, the market was a location for commercial and social hub for the Maltese. The functioning of the *Pitkali* market was dependent on a *pitkal*, a vegetable market supervisor, [91], who would negotiate price of produce with farmers, that would be sold to wholesalers, retailers, shop keepers or exported. The farmer would be paid the negotiated price less commission [92]. Each *pitkal* specialized in different produce and they came from all over the island to redistribute goods back in their home towns; according to the 1911 census there were eight produce vendors form Birchicara and eight from Curmi, and many others (number of producer vendors are provided in brackets) from Valletta (15), Floriana (13), Hamrun (8), Sliema (18), St. Julians (2), Rabato (3), Gargur (3), Lia and Attard (3), Zebbug (10), Cospicua (5), Zeitun (3), Tarxen (1), Asciak (1), and Zurrico (5) [79].

Although the *pitkal* were licensed, it was not uncommon for them to underpay farmers for their produce because farmers were not necessarily aware of the value of their goods and because there were not any regulations to control inflation [92,93].

Notwithstanding the complexities of the inner workings of the *pitkali*, the market was a deeply culturally rooted establishment in Birchicara and Maltese culture. The physical hub of the market allowed for daily person-to-person meetings between *pitkal* from various towns across the island; farmers from rural locales; and the opportunity for the pathogen to enter Birchicara and thereafter disperse to other locations.

### The railway and workmen

**Fig 7:**
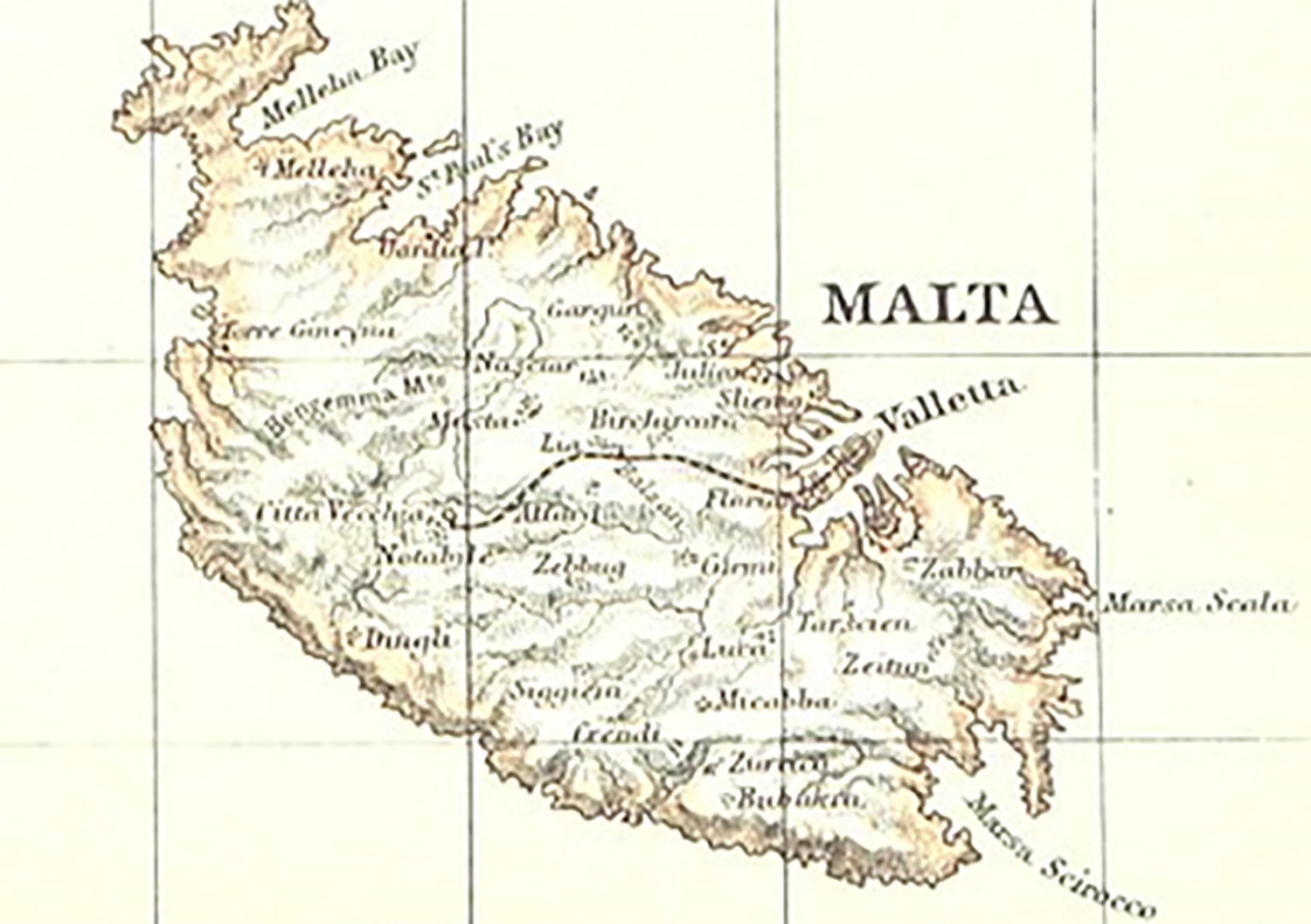
Map showing train line in Malta, (this map is copyright free and can be located at: https://commons.wikimedia.org/wiki/File:Map_of_Maltese_Islands_and_Valetta_%281888%29.jpg).

While limited in scale, Malta’s railway system played a vital role in commercial activities, as well as the movement of people and military personnel and the sick and wounded during the war years. Known by the locals as ‘il-vapur tal-art’, the ship on land, [94], the train line connected Valetta to the former capital center of Mdina. The railway consisted of 11.1 km of track with twelve stops, connecting about 38 percent of the population on the island and was in operation from 1883 until 1931 (Fig 7).

As the Central Station, Birchicara was a transfer station where trains from both directions from Valletta to Rabat and vice versa would meet. One train had a carriage with a mailbox attached to the side, where commuters could deposit their mail at the Central Station [95]. What set the Central Station apart from other stations on the train line, was that a special rate, the Workmen’s fare was available for men travelling from Valletta to Birchicara and vice versa. As described by P. Galea, the reduced fare was available for the first trains in the morning and the last train at 17:45 PM. A similar reduced fare was also available for the whole route from Mdina to Valletta, and there were workmen carriages on the train. These carriages, although open air design, would have facilitated the spread of the influenza virus because the men were in very close contact to each other and the travelers were not wearing masks (as it was not a mitigation strategy put in place during the pandemic). The fact that Birchicara had a special rate meant that there was a concentration of working men either residing in Birchicara or from nearby locations and using the station to travel to work in other locations east of Birchicara, such as the dockyards in Valletta. In fact, Birchicara along with two other cities have been described as the harbours’cornubation [96]. The daily movement of men to-and-fro the dockyards was a constant, as it was the single most important source of employment during the war on the island. For those residing in an urban city, a man would simply walk to work given that there was no local affordable public transportation system in place. In contrast, workmen residing in the surrounding suburban areas were dependent on the railway for transportation to the workplace. As a consequence, the suburbs were attractive locations for immigration since 1861, (even prior to the railway) especially for those residing in the overpopulated urban centres [80], as well as for those moving from rural towns. The population of suburbs also increased because of natural growth [96]. In 1842, 15 percent of the population resided in suburban towns, and by 1921, the suburban population totaled 39 percent [96]. In Birchicara alone, between 1851 and 1901 the population increased by 2,000 individuals [96].

Ridership during the war years was high, and the system accommodated extra traffic with two locomotives and an increased number of carriages. Unlike the Maltese men who were enlisted the Military, those who worked at the dockyard as civilians could move freely from their place of employment to their homes at the end of the work day. The Maltese in the military required sleeping out passes to return home at the end of day during the war, but during the height of the pandemic the passes were canceled [57]. It is very likely that the Birchicara men working at the dockyard in Valletta could have carried the virus back with them to their household. As a case in point, during the second wave of the epidemic, the 1918/19 influenza mortality was 0.8 per 1000 per living for communities along the train route and 0.58 per 1000 for settlements not on the route. The difference was statistically significant (Z-score = 2.262, p = 0.024).

The other mode of transportation, the tram, which connected some communities along the train line such as Birchicara, Harum, Floriana, and Valletta, as well as other communities off the line: Zebbug, Curmi, Paola, Copsicua, Vittoriosa, was not operating during the First World War, beginning on Oct 10^th^, 1917, because of the lack of spare parts and shortage of the supply of coal needed to fuel the trams [97]. The tram resumed service on June 27^th^, 1919. Further, motor buses were not available in Malta until after 1920 [94].

### The 1916/17 measles epidemic and inseparability with the British military

Given that Birchicara had the highest rates of measles (during the 1916/17 epidemic) and whooping cough (in 1917/18), we proposed that these two diseases may have had the potential to contribute to the elevated influenza morbidity and mortality via an immunological or scarring process.

Measles is known to impact on the innate immune memory of children, by diminishing pre-existing antibodies that offer protection from other pathogens, by causing immunological immaturity or compromising the immune memory to previous encountered pathogens. Moreover, the literature supports a measles induced immune suppression that can last up to 3 years after the initial infection because the virus infects memory T lymphocytes, resulting in apoptosis and a prolonged state of immune suppression [49]. Consequently, children are placed at a higher risk of infection with respiratory diseases [39,40].

Similarly, it has been reported that whooping cough may increase risk of or severity of subsequent infection of respiratory disease (such as influenza) [98,99], perhaps via toxin-mediated suppression of innate immunity [100]. However, we did not find a statistically significant relationship between whooping cough deaths and childhood influenza deaths.

In the case of measles, epidemics occurred when there were enough susceptible children [101]. The inseparable nature of the daily lives of the Maltese and British Forces stationed on the island facilitated the spread of disease. As a case in point, Dr. Hennen, the Chief Medical Officer of Health, commented that during the 1824 measles epidemic, the disease was imported to the island by the 95^th^ Regiment, then into the hospital of the 80^th^ Regiment whereby it was transmitted to families of the regiment, eventually infecting children in the civilian population [102].

At the beginning of the 1918 pandemic, the First World War was still ongoing, many communities hosted British Military bases and/or a makeshift military hospitals. The presence of British Military within the various suburban and urban communities was not an uncommon sight, and the civilians benefited from the opportunity to gain part- or full-time employment through supporting the needs of the military personnel. The military bases were where food stuffs that was in short supply could be had by the civilian population. What set suburban communities apart was the fact that garrison women and presumably their children resided in these towns [92], most likely because locations such as Birchicara were more affordable and offered spacious accommodations, while the husbands were encamped in the fortified cities.

In 1918, Birchicara was described as having Military barracks (St. Patrick’s), which was also a hospital [103], and in 1911 there were three barracks and and/or Military quarters [79]. By the First World War there was also an active airbase, Saint Sebastian. The movement of British Forces personnel in and out of the town, and the families of these personnel residing and mingling with civilians could have contributed to the spread of the disease, including pandemic influenza in Birchicara.

### A syndemic potential

We propose that similar to the syndemic interaction of respiratory tuberculosis with pandemic influenza in Malta [58], that there was a syndemic of the 1916 to 1917 measles epidemic with 1918 influenza mortality in children in Birchicara.

We argue that in addition to the interaction of the two infectious diseases, that the market place and train hub, along with the abysmal living conditions, poverty, and unemployment created the opportunity for a syndemic to occur and could explain the heightened mortality of 1918childhood influenza mortality observed in suburban communities, and in particular in Birchicara.

During the turn of the century, Birchicara was described in unflattering terms; as such the Annual Health Report described the living conditions in 1909 as deplorable:

Birchicara was a large, populated centre in the Valetta sub-urban area. Estimated population (middle of 1909–10) 9554 mostly of the artisan class. It is in a hollow, at low elevation: number of dwelling (separate houses, rooms and kerrejas) [are] 1971; old buildings prevail, with damp grounded-floors and inadequate window space, a good many have trapped closet basins, handed-flushed with few exceptions, of which some drain to cesspools, some to a new branch sewer, the majority to old branch sewers, all linked with the main sewage system of the Island. In undrained dwellings and removed to the fields at irregular intervals. The condition of the old sewers and the house drains is not satisfactory.

Lia (Lia, Balzan and Attard) practically one large rural populated center of which Balzan is now in contact with Birchicara by mutual expansion. … not so overcrowded as Birchicara, generally more healthy. …

The same remarks apply to the disposal of night soil in undrained dwelling and others as in Birchicara. [101] (p. J25)

Commenting on the effectiveness of the mitigation measures that were put in place during the 1918 pandemic at the time (such as sanitizing of public places), the Principal Medical Officer of Health, Dr. Bernard, noted that the citizens of Bircchicara (known as the karkariz), as well as the inhabitants of the nearby communities of Curmi and Zeitun, as being so impoverished that there were not any cinema theaters, nor were the inhabitants a class of people who would frequent the cinema [104].

Although conditions in Birchicara were not conducive to adequate healthy living conditions, and with much of the islandbeing impoverished, we argue that the traditional factors that have typically contributed to increased influenza rates, and which were usually byproducts of urban settlements: high overcrowding rates and the related proxy measure of poor health: infant mortality [105,106], did not appear to explain the higher rate of influenza mortality in Birchicara, because Birchicara did not display extremely high rates for the aforementioned factors.

Similarly, a large-scale study on American counties have found counter intuitive relationships of population density with COVID-19 rates [107]. In over 1000 metropolitan counties across the USA, large metropolitan size (measured in terms of population) led to significantly higher COVID-19 infection rates and higher mortality rates. Thus, metropolitans with large areas (sprawling areas) and a higher number of counties tightly linked together through economic, social, and commuting relationships, were the most vulnerable to the pandemic outbreaks [108]. County density was related to significantly lower infection COVID-19 rates and lower death rates [107]. These findings suggest that the relationship between density and the pandemic is more complex than a simple correlation. Even though population density theoretically increases the concentration of people and facilitates person-to-person contact, density may lead to a better (perhaps more centralized) health care infrastructure that was more equipped to respond to the pandemic, as well as more effective at implementation of social distance policies and practices [108].

### Conclusion

Studies on epidemics and pandemics of the past have largely ignored the experience in the suburbs. This paucity in research is probably because information on suburban communities is non-existent, or lumped together with urban communities.

In nations with a history of widespread poverty and numerous epidemics of infectious disease, (especially measles), ‘traditional’ risk factors such as high population density and crowding may not explain high rates of infectious disease. This may also be the case for contemporary societies that are heterogenous with respect socio-economic status. Instead, movement of people via crowded trains or planes, to and from hubs (such as a marketplace) may be more important for facilitating the spread of infection and mortality, as has been observed in suburban locations where major transportation hubs (airports) are located during the COVID-19 pandemic. The impacts of the COVID-19 pandemic are accelerating the “suburbanisation of inequality,” because the more vulnerable workers who primarily reside in the lower cost suburbs are expected to travel during lockdowns to their low wage and part-time work [109].

## Supporting information

S1 Dataset 1918/19 influenza death information by settlement for Malta. (XLSX)

S1 Striking image Folk medicine in Gozo (photo courtesy of L. A. Sawchuk). (TIF)
