# Supplementary figures and images for: The emergence of a suburban penalty during the 1918/19 influenza pandemic in Malta: The role of a marketplace, railway, and measles
Source: PLOS Glob Public Health. 2023 Sep 1;3(9):e0002167. doi: 10.1371/journal.pgph.0002167 (PMC10473495; doi:10.1371/journal.pgph.0002167)

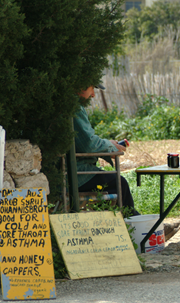

Supplement: S1 Striking image — (TIF) [file pgph.0002167.s002.tif]
